# Talking About Incarceration History: Engaging Patients and Healthcare Providers in Communication

**DOI:** 10.1007/s11606-024-09149-z

**Published:** 2024-10-31

**Authors:** Ankita Patil, GeorgePatrick J. Hutchins, Harika Dabbara, Veronica L. Handunge, Annie Lewis-O’Connor, Rahul Vanjani, Monik C. Botero

**Affiliations:** 1 Division of Women’s Health, Department of Medicine, Brigham and Women’s Hospital, Boston, MA USA; 2 Harvard Medical School, Boston, MA USA; 3 Department of Sociology, Harvard University Graduate School of Arts & Sciences, Cambridge, MA USA; 4 Boston University Aram V. Chobanian & Edward Avedisian School of Medicine, Boston, MA USA; 5 Department of Social and Behavioral Sciences, Harvard T.H. Chan School of Public Health, Boston, MA USA; 6 Department of Medicine, Warren Alpert Medical School of Brown University, Providence, RI USA; 7 Department of Epidemiology, Harvard T.H. Chan School of Public Health, Boston, MA USA

**Keywords:** incarceration, patient care, health equity, social determinants of health

## Abstract

**Background:**

Incarcerated individuals in carceral facilities demonstrate an elevated prevalence of chronic disease conditions which are likely to persist post-release. Healthcare providers may not be trained on how exposure to incarceration may influence patient health outcomes and patient-provider communication.

**Objective:**

To examine the self-perceived preparedness of healthcare providers to interview patients regarding history of incarceration and the potential related health consequences.

**Design:**

This cross-sectional study consisted of a web-based self-administered questionnaire distributed via email to a random sample of healthcare providers in the Department of Medicine at Brigham and Women’s Hospital.

**Participants:**

In total, 400 healthcare providers were invited to participate; 114 respondents completed the survey, of which 26% were medical doctors (*n*=30), 41% were physician assistants (*n*=47), and 32% were nurse practitioners (*n*=37).

**Main Measures:**

Understanding healthcare provider training in caring for formerly incarcerated patients, current treatment practices and confidence caring for patients who have experienced incarceration, and implications for clinical care.

**Key Results:**

Of 114 respondents, 73% reported that they currently care for formerly incarcerated patients. However, only 8% received specialized training for the care of formerly incarcerated patients. While most respondents did not ask their patients about prior history of incarceration (81%), when asked about comfortability in doing so, 60% reported low levels of comfort. Most providers (77%) reported high agreement that incarceration impacted health, with 54% reporting that it led to significant healthcare access barriers, but 64% reported low confidence levels in addressing the needs of formerly incarcerated patients.

**Conclusions:**

Healthcare workers recognized incarceration as a detrimental health exposure. However, providers reported low levels of confidence in understanding and addressing the unique needs of patients who experienced incarceration. Findings support the need for further training regarding how to address the needs of formerly incarcerated patients, which would support efforts towards achieving equitable healthcare.

## INTRODUCTION

The American Public Health Association has recognized incarceration as a profound social determinant of health (SDOH), intersecting with and further exacerbating other SDOHs such as poverty, racism, and limited access to healthcare. The unique entrenchment of the carceral system within the USA and disproportionate burden among historically marginalized communities requires a tailored response to its individual, intergenerational, and community level consequences.^1^ Incarcerated populations and those with a history of incarceration disproportionately suffer from chronic diseases, mental health issues, and substance use disorders compared to the general population.^2^ This increased burden of disease is not simply a byproduct of incarceration but is exacerbated by systemic issues experienced during incarceration such as overcrowding, infectious disease, inadequate medical care, and disrupted treatment continuity.^3–5^

Yet these determinants continue to severely impact individuals upon re-entry, and often transition into increasingly complex challenges. Presence of incarceration-related ethno-racial inequities in health remains an intractable problem rooted in systemic racism.^6^^,^^7^^,^^8^ The long-term ramifications of incarceration further include punitive civil penalties and pervasive cultural stigma, which collectively impede the utilization of health and social services, promote systems avoidance, and exacerbate health inequities among populations exposed to incarceration.^9,10^ For example, formerly incarcerated individuals face a heightened risk of mortality and face substantial barriers in accessing housing, food security, and employment. These systemic barriers are compounded by significant challenges in securing healthcare services and insurance, which are critical upon re-entry. These challenges to securing fundamental health and wellness needs are further complicated by the enduring systemic racism in the USA which is overtly manifested in the racial and ethnic inequities characteristic of the US carceral system.

Despite the longstanding history of incarceration in the USA and associated challenges faced by individuals and communities most directly impacted, healthcare providers often lack specific training on how to effectively care for patients who have experienced incarceration. Research has demonstrated a critical gap in the education and preparation of healthcare workers, which can affect the patient-provider relationship and subsequent health outcomes. This gap underscores the need for educational programs that equip healthcare providers with the knowledge and skills necessary to effectively meet the unique health needs of formerly incarcerated individuals.

To address these gaps, our study aims to examine the self-perceived preparedness of healthcare providers at Brigham and Women’s Hospital (BWH) Department of Medicine (DOM) to interview patients regarding their history of incarceration and address the health-related consequences. We additionally seek to inform healthcare providers and educators regarding the need to integrate a more comprehensive public health approach into the education and training of our healthcare workforce to address the needs of this historically marginalized population, advocating for health equity and justice as essential components of healthcare delivery.

## METHODS

We aimed to capture healthcare providers’ perceptions with respect to caring for patients with a history of incarceration by conducting a cross-sectional survey of providers from three professional tracks (medical doctor (MD), physician assistant (PA), nurse practitioner (NP)). Due to a lack of an established instrument, we developed a novel survey instrument to examine healthcare providers’ training history with respect to carceral health, patient-provider communication, and provider perceptions of incarceration implications on clinical care. The instrument was developed iteratively for content validity with clinicians and community health workers with direct experience providing care for formerly incarcerated patients. The instrument was then piloted among 21 healthcare professionals across the three identified professional tracks. Extensive revisions were incorporated, resulting in a final instrument with close- and open-ended responses administered through REDCap as a 10–15 min web-based questionnaire.

A list of providers and their emails in the Department of Medicine at Brigham and Women’s Hospital (BWH) was obtained from the employee directory available to all BWH employees. A random sample of participants stratified by professional role (e.g., physicians, physician assistants, and nurse practitioners) was selected. An equal number of physicians, physician assistants, and nurses were then selected to make up the total of 400 providers invited to participate. Because the list of physicians included both clinical and research-only faculty, research faculty were removed from the sample. The survey, which was anonymous and self-administered, was sent via email with a description of the study to all selected participants. From the 400 invited participants, we aimed to recruit 60 responses from each professional role, for a total of 180 participants. Study “Champions” from each professional role were selected to describe the study and encourage their colleagues to participate if randomly selected to participate. “Champions” were added as study staff and did not fill out the survey themselves. Participants were compensated with a $15 gift card upon survey completion. The survey can be found in the Appendix.

### Analysis

Responses were analyzed across all respondents and by profession using descriptive statistics and were reported as frequencies or relative frequencies. Responses collected on 5-point Likert scales were categorized as low (do not/slightly), moderate (moderately), or high (very strongly/extremely). We used Fisher’s tests and chi-squared tests, as appropriate based on sample size, with *alpha* < .05 to determine statistical significance when comparing across professions. However, it should be noted that statistical testing for differences across professions was not the primary goal of this study but is provided for contextualization. The study protocol was deemed exempt by Mass General Brigham Institutional Review Board (IRB). All quantitative data generated during this study are recorded in Tables 1, 2, and 3.

**Table 1:** Participant Characteristics and Education

	Total sample (*N*=114)	Medical doctor (*n*=30)	Physician assistant (*n*=47)	Nurse practitioner (*n*=37)	*P* value
Formal training for patients currently in correctional facility^*^					
Yes	11 (10)	5 (17)	2 (4)	4 (11)	0.19
Formal training for formerly incarcerated^†‡^					
Yes	9 (8)	5 (17)	1 (2)	3 (9)	0.07
Importance of training^§^					
Slightly important	12 (11)	2 (7)	6 (13)	4 (11)	0.19
Moderately important	43 (38)	9 (30)	23 (49)	11 (30)	
Very/extremely important	59 (52)	19 (63)	18 (38)	22 (60)	
Gender^‖^					
Male	22 (20)	14 (48)	7 (15)	1 (3)	< 0.001
Female	90 (80)	14 (48)	40 (85)	36 (97)	
Do not want to answer	1 (1)	1 (4)	0 (0)	0 (0)	
Race/ethnicity					
American Indian/Alaskan Native	3 (3)	1 (3)	1 (2)	1 (3)	0.68
Asian	15 (13)	6 (20)	6 (13)	3 (8)	
Black/African American	3 (3)	1 (3)	1 (2)	1 (3)	
Native Hawaiian/Pacific Islander	0 (0)	0 (0)	0 (0)	0 (0)	
Hispanic, Latino/a/x, Spanish origin	5 (4)	1 (3)	1 (2)	3 (8)	
White	95 (83)	22 (73)	40 (85)	33 (89)	
Do not want to answer	3 (3)	2 (7)	0 (0)	1 (3)	
Personal, family, or friends’ incarceration					
Myself	0 (0)	0 (0)	0 (0)	0 (0)	0.17
Family member or friend	23 (20)	7 (23)	9 (19)	7 (19)	
None	91 (80)	23 (77)	38 (81)	30 (81)	

^*^“Unsure” response dropped

^†^Missing two responses (denominator = *n*-2)

^‡^No responses for “Unsure”

^§^No responses for “Not at all important”

^‖^Missing one response (denominator = *n*-1)

**Table 2:** Patient-Provider Communication

	Total sample (*N*=114)	Medical doctor (*n*=30)	Physician assistant (*n*=47)	Nurse practitioner (*n*=37)	*P* value
Currently treating formerly incarcerated patient					
Yes	84 (73)	27 (90)	31 (66)	26 (70)	0.07
Unsure	20 (18)	1 (3)	10 (21)	9 (24)	
Comfort asking prior history					
Not at all comfortable	23 (20)	4 (13)	9 (19)	10 (27)	0.40
Slightly comfortable	45 (40)	12 (40)	20 (43)	13 (35)	
Moderately comfortable	31 (27)	12 (40)	12 (26)	7 (19)	
Very/extremely comfortable	15 (13)	2 (7)	6 (13)	7 (19)	
Provider initiated ask of history of incarceration^*^					
Yes	21 (19)	11 (37)	6 (13)	4 (11)	0.02
If answered “yes” to the previous question, how often?					
Routinely asked	5 (24)	1 (9)	1 (17)	3 (75)	0.14
Sometimes asked	7 (33)	4 (36)	2 (33)	1 (25)	
Rarely asked	9 (43)	6 (55)	3 (50)	0 (0)	
If answered “yes” to the previous question, asked for reason for incarceration					
Yes	3 (14)	0 (0)	1 (17)	2 (50)	0.06
Unsure/certain circumstances	3 (14)	1 (9)	2 (33)	0 (0)	
If answered “yes” to the previous question, documentation after asking about reason for incarceration					
Document in note	3 (100)	0 (0)	1 (100)	2 (100)	N/A
Document elsewhere in EMR	0 (0)	0 (0)	0 (0)	0 (0)	
Reason given by patient, document information in electronic medical record^*^					
Yes	28 (25)	4 (13)	14 (30)	10 (28)	0.26
Unsure	54 (48)	16 (53)	24 (51)	14 (39)	
Reason given by patient, honor request to not document					
Yes	48 (42)	16 (53)	18 (38)	14 (38)	0.18
Unsure	52 (46)	8 (27)	25 (53)	19 (51)	
Only under certain circumstances	11 (10)	4 (13)	3 (6)	4 (11)	
Increased concern for personal safety when treating formerly incarcerated patient^†^					
Do not agree	61 (54)	16 (53)	28 (60)	17 (46)	0.24
Slightly agree	34 (30)	6 (20)	13 (28)	15 (41)	
Moderately agree	17 (15)	8 (27)	5 (11)	4 (11)	
Very strongly agree	2 (2)	0 (0)	1 (2)	1 (3)	
Need to know reason for incarceration to feel safe when treating patient^*^					
Do not agree	59 (52)	17 (57)	27 (59)	15 (41)	0.43
Slightly agree	34 (30)	9 (30)	11 (24)	14 (38)	
Moderately agree	14 (12)	3 (10)	7 (15)	4 (11)	
Very strongly/extremely agree	6 (5)	1 (3)	1 (2)	4 (11)	

^*^Missing one response (denominator = *n*-1)

^†^No responses for “Extremely agree”

**Table 3:** Implications for Clinical Care

	Total sample (*N*=114)	Medical doctor (*n*=30)	Physician assistant (*n*=47)	Nurse practitioner (*n*=37)	*P* value
Better care given knowing patient’s history^*^					
Yes	71 (63)	21 (70)	27 (58)	23 (64)	0.86
Unsure	34 (30)	7 (23)	16 (34)	11 (31)	
Greater trust if patient’s history is known^*^					
Yes	68 (60)	18 (60)	27 (58)	23 (64)	0.95
Unsure	35 (31)	9 (30)	15 (32)	11 (31)	
Confidence level in addressing formerly incarcerated patient’s needs					
Not confident	34 (30)	7 (23)	15 (32)	12 (32)	0.88
Slightly confident	39 (34)	10 (33)	15 (32)	14 (38)	
Moderately confident	34 (30)	10 (33)	15 (32)	9 (24)	
Very/extremely confident	7 (6)	3 (10)	2 (4)	2 (5)	
Confidence level in referring formerly incarcerated patient					
Not confident	32 (28)	6 (20)	16 (34)	10 (27)	0.81
Slightly confident	21 (18)	8 (27)	7 (15)	6 (16)	
Moderately confident	32 (28)	9 (30)	12 (26)	11 (30)	
Very/extremely confident	29 (25)	7 (23)	12 (26)	10 (27)	
Level of agreement: “If a patient shared with me that they had experienced incarceration, I'm confident that I would understand the consequences that experience may have on their life.”^†^					
Do not agree	41 (36)	6 (20)	20 (43)	15 (41)	0.25
Slightly agree	39 (34)	13 (43)	16 (34)	10 (27)	
Moderately agree	26 (23)	10 (33)	8 (17)	8 (22)	
Very strongly agree	8 (7)	1 (3)	3 (6)	4 (11)	
History of incarceration considered in treatment plan					
Yes	73 (64)	20 (67)	28 (60)	25 (68)	0.94
Unsure	29 (25)	7 (23)	13 (28)	9 (24)	
If answered “yes” to the previous question, confidence level in incorporating information in treatment plan					
Not confident	20 (27)	4 (20)	8 (29)	8 (32)	0.98
Slightly confident	20 (27)	6 (30)	7 (25)	7 (28)	
Moderately confident	24 (33)	7 (35)	10 (36)	7 (28)	
Very/extremely confident	9 (12)	3 (15)	3 (11)	3 (12)	

Incarceration impacts patient’s health^*‡^					
Slightly agree	4 (4)	0 (0)	3 (7)	1 (3)	0.64
Moderately agree	22 (20)	5 (17)	8 (17)	9 (24)	
Very strongly/extremely agree	87 (77)	25 (83)	35 (76)	27 (73)	
Health conditions affected by incarceration					
Arthritis	35 (31)	12 (40)	15 (32)	8 (22)	0.26
Cancer	44 (39)	13 (43)	15 (32)	16 (43)	0.47
CVD	79 (69)	22 (73)	30 (64)	27 (73)	0.57
Chronic pain	81 (71)	25 (83)	30 (64)	26 (70)	0.18
Cognitive decline	72 (63)	24 (80)	28 (60)	20 (54)	0.07
Diabetes	57 (50)	18 (60)	24 (51)	15 (41)	0.28
Infectious disease	82 (72)	24 (80)	30 (64)	28 (76)	0.25
Mental health (substance use)	103 (90)	28 (93)	41 (87)	34 (92)	0.63
Mental health (other)	98 (86)	28 (93)	38 (81)	32 (87)	0.31
Obesity	52 (46)	18 (60)	21 (45)	13 (35)	0.13
Reproductive health	34 (30)	10 (33)	12 (26)	12 (32)	0.70
Rheumatic disease	20 (18)	6 (20)	10 (21)	4 (11)	0.41
Sexually transmitted disease	81 (71)	26 (87)	32 (68)	23 (62)	0.08
Total mortality	79 (69)	23 (77)	27 (58)	29 (78)	0.07
Other	4 (4)	2 (7)	0 (0)	2 (5)	N/A
Unsure	6 (5)	1 (3)	3 (6)	2 (5)	N/A
Formerly incarcerated patient’s face significant barriers to accessing care^‡^					
Slightly agree	11 (10)	3 (10)	7 (15)	1 (3)	0.35
Moderately agree	42 (37)	9 (30)	17 (36)	16 (43)	
Very strongly/extremely agree	61 (54)	18 (60)	23 (49)	20 (54)	
Formerly incarcerated patient’s face difficulty adhering to medication/other treatments					
Do not agree	5 (4)	1 (3)	2 (4)	2 (5)	0.51
Slightly agree	24 (21)	5 (17)	14 (30)	5 (14)	
Moderately agree	45 (40)	15 (50)	16 (34)	14 (38)	
Very strongly/extremely agree	40 (35)	9 (30)	15 (32)	16 (43)	

^*^Missing one response (denominator = *n*-1)

^†^No responses for “Extremely agree”

^‡^No responses for “Do not agree”

## RESULTS

### Participant Characteristics and Education

In total, 114 respondents from a large urban health center completed the survey, of which 26% were MDs (*n*=30), 41% were PAs (*n*=47), and 32% were NPs (*n*=37). While the majority of PAs and NPs identified as female (85% and 97%, respectively), approximately half of MDs identified as female (48%). The majority of participants identified as White (83%) irrespective of professional role (*n*=95). None of the respondents had experienced incarceration themselves, and 80% reported they did not have a family or friend who had been incarcerated. Notably, 92% of participants reported that they had not received any formal training on caring for patients with a history of incarceration, with more MDs (17%) reporting to have received formal training than PAs (2%) and NPs (9%). Yet, 73% of all respondents reported that they currently provided treatment for patients with a history of incarceration, including 90% of MDs that responded (Table 2). Drawing from the open-ended responses, two participants noted providing medical care within a correctional facility. These experiences included outpatient general medicine and specialist care, as well as inpatient psychiatric services. One respondent noted prior work in a county hospital setting where they regularly took care of patients from the local jail. Over half of all MD and NP respondents (63% and 60%, respectively) felt that being trained to care for formerly incarcerated patients was of high importance, compared to just 38% of PAs. Additional participant characteristics are noted in Table 1.

### Patient-Provider Communication

Most respondents reported that they did not ask their patients about prior history of incarceration (81%). Among the minority of respondents that reported asking, less than a quarter “routinely” asked and 43% “rarely” asked. Text responses show that one of the most common reasons for asking about this prior history is to identify risk factors for infectious diseases, most prominently tuberculosis. Other respondents noted obtaining this information as either part of a routine social history or standard screening tool or as the result of seeing information about a history of incarceration documented in another provider’s note. When sharing comfort levels in asking patients about history of incarceration, 60% of respondents were either “not comfortable” or “slightly comfortable.” If the reason for incarceration was shared, 48% of respondents reported being “unsure” if they would document it in the patient’s electronic medical record (EMR). If the patient provided the reason for incarceration and further asked it to not be documented in their EMR, more PAs and NPs were “unsure” than MDs if they could honor this request (MD, 27%; PA, 53%; NP, 51%); approximately half of MD respondents (53%) stated they would be able to honor this request. Open-ended responses reveal that much of the uncertainty surrounding ability to honor such a request was related to issues such as relevance to that patient’s medical care and type of offense committed. One respondent noted that they would document further information about history of violent crime and sexual offenses, noting that the latter reflects information that is “more public.” While over half of respondents from the MD (53%) and PA (60%) groups reported that they did not feel an increased concern for personal safety when treating formerly incarcerated patients, 55% of NPs reported that they did feel an increased concern. A similar pattern was noted when respondents considered whether they need to know the reason for a patient’s incarceration to feel safe when treating them; compared to 41% of NPs, 57% of MDs and 59% of PAs felt that they did not need to know the reason for a patient’s incarceration to feel safe when treating them. Further questions related to patient-provider communication and the respective responses are noted in aggregate and by profession in Table 2.

### Implications for Clinical Care

We also asked providers to report their confidence in understanding the types of unique exposures patients with a history of incarceration may face and their perception of how knowing about a patient’s history of incarceration may inform their clinical care. Most providers (overall 77%; MD, 83%; PA, 76%; NP, 73%) reported high agreement that incarceration impacted health and 54% (MD, 60%; PA, 49%; NP, 54%) believed it led to significant healthcare access barriers. Additionally, 35% of respondents reported high agreement that this patient population may face difficulty adhering to medication and other treatments. When given a list of health conditions, of which providers were asked to state whether these conditions were affected by incarceration or not, most providers reported this patient population suffers from substance use disorder (90%) and other mental health conditions (86%). Other health conditions that most providers noted formerly incarcerated patients face include infectious disease (72%), chronic pain (71%), sexually transmitted diseases (71%), total mortality (69%), and cardiovascular disease (69%). Open response answers included additional mentions of specific respiratory conditions such as asthma and chronic obstructive pulmonary disorder (COPD) and psychiatric conditions such as post-traumatic stress disorder (PTSD).

Among survey respondents, 63% of providers reported that better care could be provided if they are aware of the patient’s history and that greater trust could also be established (60%). While 64% of respondents reported that they would consider history of incarceration in their treatment plan and considered it important, within this group, 54% were “not confident” or only “slightly confident” in their ability to incorporate this history into their plan. Furthermore, an overall 64% (MD, 56%; PA, 64%; NP, 70%) reported low confidence levels in addressing the needs of patients with a history of incarceration. When asked about their level of agreement that they would understand the consequences incarceration may have on the patient’s life, 36% did not agree, indicating the need for further education on the health impacts of incarceration and associated treatment consequences when caring for formerly incarcerated patients. Additional questions related to clinical care implications and the respective responses are noted in aggregate and by profession in Table 3.

## DISCUSSION

Our study revealed disconcerting patterns among healthcare providers (MDs, PAs, and NPs) of low self-perceived preparedness and confidence in caring for formerly incarcerated patients. These data are the first to our knowledge to survey healthcare providers regarding their self-reported confidence in treating patients with a history of incarceration. Findings expose a need for clinical training and highlight the importance of including this education to address the unique needs of this vulnerable population.

Firstly, our findings point to the deficit in incorporating carceral health in clinical training, as only 18% of physicians in our sample reported to have received training on carceral health during their medical school education. This finding is further corroborated by existing literature; there is a paucity of data regarding clinical training in carceral health in medical, physician assistant, and nursing programs. The only available data is limited to medical schools’ curriculums, which indicate that only 20% offer this training and 50% offer rotations at sites, of which 38% were elective^11^. This absence of foundational knowledge has palpable consequences for supporting equity in clinical practice and underscores the need to equip all healthcare providers with the proper tools to meet the urgent needs of carceral-involved patients. Not only are discussions critical, but efforts from all healthcare providers are necessary to interrupt the cycles of oppression and barriers to accessing medical care perpetuated by those involved in managing and caring for incarcerated individuals.

Furthermore, the lack of actionable guidance for healthcare professionals on navigating conversations with formerly incarcerated patients is apparent. While the clinicians in our sample acknowledged the significant impact a history within the legal punishment system has on overall health and expressed intent to accommodate the unique needs of incarcerated patients, they reported receiving minimal guidance on how to incorporate this information into clinical decision-making. For example, the scenario of whether to honor a patient’s request to not document their carceral history showcased this disconnect between well-intentioned providers and their actionable knowledge. Therefore, the limitations in training and guidance may not only impair clinical acumen but also hinder the ability to deliver ethical and patient-centered care, which further perpetuates existing health inequities.

This underlines the need for targeted training on how to utilize non-stigmatizing methods to screen for incarceration history–related health risks and incorporate this pivotal information into a treatment plan. Given the sensitivity of this topic and the discomfort some providers may feel when asking about incarceration history, providers would benefit from training on appropriate verbal and non-verbal communication to use when interviewing patients and how to protect patients’ privacy in medical record documentation. It would be critical for a substantial portion of this training to involve individuals directly impacted by incarceration, who can work with providers to develop appropriate language and interviewing techniques. Additionally, a trauma-informed approach focused on developing rapport and addressing patient needs without re-traumatization would be instrumental in providing equitable healthcare. Existing studies provide a roadmap for implementing trauma-informed approaches for carceral involved populations; for example, this can be done by centering the roles of safety, hope, autonomy, respect, and empathy (SHARE) when interacting with patients and providing comprehensive education on the demographic distribution of various forms of trauma. The potential benefits of such training were recognized by several providers in our own survey, who, in their open-ended responses, cited specific clinical examples of how additional guidance on how to handle encounters involving incarcerated patients would enhance therapeutic effectiveness. Educating healthcare providers regarding how a history of incarceration may adversely impact health through intersections with other social determinants of health is also imperative. For example, a Criminal Offender Record Information (CORI) can impede employment, education, and access to housing. Furthermore, clinicians can be empowered to leverage their unique roles to advocate for their formerly incarcerated patients through a multitude of ways, such as by protecting and facilitating their patient’s ability to remain in their home communities through holistic approaches to meet patients’ physical and mental health needs^12^. Importantly, these discussions and educational gaps should be offered early and at multiple timepoints in a clinician’s journey, with incentives such as continuing medical education (CME) credit being offered as a means of institutionally affirming their importance.

The Academy of Family Physicians (AFP) outlines several constraints and challenges that physicians may face when caring for incarcerated populations, including specific areas of concern for the care of formerly incarcerated patients, such as the transfer of medical records, limited access to dental and eye care, and a notable lack of continuity of care upon release^13^. Additionally, research by Haber et al. (2019) underscores the nuanced issues faced in treating this demographic, particularly emphasizing the importance of clinical considerations, such as the use of shackling, protecting patient privacy, and the disclosure of discharge plans^14^. These elements are crucial not only within correctional settings, but also significantly impact the approach to healthcare for patients post-release. Consideration is also given to the implications surrounding care of incarcerated patients outside of correctional settings, given the constitutional right to healthcare accorded to this population via the Eighth Amendment. While the primary motivation for ensuring adequate training for medical professionals treating this group should remain patient-centered, the potential legal ramifications of administering inadequate care due to the unfamiliarity of best practices cannot be lost. This holistic approach acknowledges the transition of care needs from incarceration to release and the importance of preparing healthcare professionals to meet these needs effectively, thus bridging the gap in care continuity and addressing the broader health disparities faced by this population.

Here, we outline a path forward with regard to improving upon providers’ confidence in providing care to this population. First, we call for early exposure to the nuances of carceral health at the beginning of one’s education in the form of patient clinics and group discussion with formerly incarcerated patients willing to speak on their experiences. This can and should be reinforced throughout a student’s education, involving direct clinical experiences with the incarcerated population as a means to develop precise language and patient-centered communication skills. Second, discussions related to social determinants of health tend to center dimensions such as socio-economic status, education, and housing, oftentimes leaving incarceration virtually ignored. Incarceration must be included in these discussions, with critical attention being paid to the dynamic way in which it can have both individual, familial, and community effects through a racialized lens. These deleterious downstream effects have been well-documented in the health literature, particularly as it pertains to post-incarceration release^2,15^.

### Limitations

This study has several limitations. First, we recognize the limited generalizability of this study given the sample came exclusively from Brigham and Women’s Hospital’s Department of Medicine. We focused on this specific cohort because this study was initially funded as a pilot project to examine ways in which the patient experience can be improved. Furthermore, the demographic composition of our sample, predominantly female-identifying, White providers in the case of PAs and NPs with little personal exposure to incarceration, may further limit generalizability. This demographic skew is particularly salient given that issues of structural racism and health equity are intrinsically connected to incarceration, which disproportionately targets historically marginalized populations along lines of race and ethnicity, socioeconomic status, sexual orientation, gender identity, and mental health conditions. In addition, due to a high proportion of female respondents, primarily among NPs and PAs, we were unable to examine whether the gender of healthcare providers may result in variability of these findings.

Second, the lack of a validated instrument necessitated the development of a new instrument, which should be examined in other clinical and research settings. Third, we had a lower than anticipated response rate. However, the consistently low levels of reported confidence among our respondents suggest that actual levels of confidence may be even lower in a more comprehensive sample of providers. Given this, we assert that despite the low response rate, the risk of systematic bias in our results is minimal. It is important to note that due to the small sample size of the study, statistically significant results should be viewed with caution. It should also be noted that this study was not explicitly designed to test differences in perceptions and preparedness among different types of healthcare providers. Rather, our goal was to describe self-reported perceptions of preparedness of healthcare providers in serving this patient population while recognizing that professional role may influence those perceptions due to specific clinical responsibilities, education, and training.

## Conclusion

Healthcare workers recognized incarceration as a detrimental health exposure. However, providers reported low levels of confidence in understanding and addressing the unique needs of patients who experienced incarceration. Findings support the need for further training regarding how to discuss and address the needs of patients who have experienced incarceration, as attempting to make such decisions without established guidelines can damage the patient-provider relationship and further stall efforts towards achieving equitable healthcare.
